# Effect of age and gender on pre-operative cardiovascular risk assessment

**DOI:** 10.1186/s13741-022-00247-2

**Published:** 2022-06-02

**Authors:** Omar Chehab, Mahmoud Eldirani, Hani Tamim, Aurelie Mailhac, Maha Makki, Habib A. Dakik

**Affiliations:** 1 Departments of Internal Medicine, American University of Beirut Medical Center, Beirut, Lebanon; 2 Biostatistics Unit, American University of Beirut Medical Center, Beirut, Lebanon

**Keywords:** AUB-HAS2 Cardiovascular Risk Index, Age, Gender, Pre-operative cardiovascular evaluation

## Abstract

**Background:**

The AUB-HAS2 Cardiovascular Risk Index is a recently published tool for pre-operative cardiovascular evaluation. It is based on six data elements: history of heart disease, symptoms of angina or dyspnea, age ≥ 75 years, hemoglobin < 12 mg/dl, vascular surgery, and emergency surgery. The objective of this study is to study the effect of age and gender on the performance of the AUB-HAS2 Index in pre-operative cardiovascular risk assessment.

**Methods:**

The study population consisted of 1,167,414 non-cardiac surgeries registered in the ACS NSQIP database. The population was stratified by age (≥ 40 and < 40 years old) and by gender (men and women). Each patient was given an AUB-HAS2 score of 0, 1, 2, 3, or > 3 based on the number of data elements s/he has. The outcome measure was all-cause mortality, myocardial infarction (MI), or stroke at 30 days after surgery.

**Results:**

The overall 30-day event rate was higher in patients ≥ 40 years compared to those < 40 years (2.5% vs 0.3%, *P* < 0.0001) and in men compared to women (2.7% vs 1.8%, *P* < 0.0001). In both age and gender subgroups, there was a gradual and significant increase in the outcome measure (death, MI, or stroke) as the AUB-HAS2 score increased: from ≤ 0.5% in those with a score of 0 to more than 15% in those with a score > 3 (*P* < 0.0001). The AUB-HAS2 Index was able to stratify risk in all subgroups into low, intermediate, and high (*P* < 0.0001). Receiver operating characteristic curves showed the AUB-HAS2 Index has very good discriminatory power in both age (area under the curve (AUC) of 0.81 and 0.78) and gender (AUCs of 0.79 and 0.84) subgroups.

**Conclusion:**

This study extends the validation of the newly derived AUB-HAS2 Cardiovascular Risk Index to different age and gender subgroups with very good discriminative power.

## Introduction

The AUB-HAS2 Cardiovascular Risk Index is a recently published tool for pre-operative cardiovascular risk assessment (Dakik et al., 2019; Dakik et al., 2020a; Dakik et al., 2020b). It was derived from a prospectively enrolled cohort of patients undergoing non-cardiac surgery at the American University of Beirut (AUB), and it was validated in a wide spectrum of surgical procedures registered in the large American College of Surgeons National Surgical Quality Improvement Program (ACS NSQIP) database (User guide for the 2016 ACS NSQIP, 2019). The index is simple to acquire and has a powerful discriminatory ability to predict cardiovascular events post non-cardiac surgery. It is based on six easily acquired data elements: history of heart disease, symptoms of heart disease (angina or dyspnea), age ≥ 75 years, anemia (hemoglobin < 12 mg/dl), vascular surgery, and emergency surgery (Table 1). The index can stratify patients undergoing non-cardiac surgery into three risk groups: low risk (scores 0–1), intermediate risk (scores 2–3), and high risk (score > 3). Age and gender are known to be important determinants of risk across a wide spectrum of medical diseases (Andersson et al., 2015; Robinson et al., 2017; Mazmudar et al., 2017). The objective of this study is to analyze the impact of age and gender on the performance of the AUB-HAS2 Index in pre-operative cardiovascular risk assessment.

**Table 1:** Elements of the AUB-HAS2 Cardiovascular Risk Index

**● H**istory of heart disease **●** Symptoms of **h**eart disease (angina or dyspnea) **● A**ge ≥ 75 years **● A**nemia (hemoglobin < 12 mg/dl) **●** Vascular **S**urgery **●** Emergency **S**urgery	

## Methods

The study population consisted of 1,167,414 non-cardiac surgeries registered in the ACS NSQIP database between 2008 and 2012 (Dakik et al., 2019; Dakik et al., 2020a; Dakik et al., 2020b; User guide for the 2016 ACS NSQIP, 2019). The population was stratified by age (≥ 40 and < 40 years old) and by gender (men and women). The performance of the AUB-HAS2 Index was compared between the two age subgroups and the two gender subgroups. There were 2644 surgeries in which gender classification was missing. Those were included in the age but not in the gender analysis. Datasets after 2012 were not included in our study because they did not capture cardiac history on patients which is one of the essential elements in the AUB-HAS2 Cardiovascular Risk Index. The ACS NSQIP is a large multicenter database that collects data on patients undergoing major surgical procedures from more than 250 participating sites on more than 150 variables, including pre-operative risk factors, intraoperative variables, and 30-day post-operative mortality and morbidity outcomes (Dakik et al., 2020b). The data is collected by trained surgical clinical reviewers at each site using a systematic sampling process and is subject to regular inter-rater reliability audits to assess its quality. Required data variables are entered via web-based data collection to the ACS NSQIP website. Surgeries are entered in the database using the International Classification of Diseases (ICD) codes. Patients under the age of 18 were excluded from the database as well as minor and transplant surgeries.

Each patient was assigned an AUB-HAS2 score of 0, 1, 2, 3, and > 3 based on the number of data elements s/he has. The AUB-HAS2 elements are history of heart disease, symptoms of heart disease (angina or dyspnea), age ≥ 75 years, anemia (hemoglobin < 12 mg/dl), vascular surgery, and emergency surgery. Patients were designated as having a history of heart disease if they had a history of prior myocardial infarction, coronary angioplasty, cardiac surgery, heart failure, atrial fibrillation, or moderate/severe valvular disease confirmed by echocardiography. The primary outcome measure was all-cause mortality, myocardial infarction, or stroke at 30 days after surgery (Dakik et al., 2019; Dakik et al., 2020a; Dakik et al., 2020b). Myocardial infarction was defined by ECG changes indicative of an acute MI (one or more of the following three: ST elevation > 1 mm in two or more contiguous leads, new left bundle branch, or new q-waves in two of more contiguous leads) or new elevation in troponin greater than three times the upper level of the reference range in the setting of suspected myocardial ischemia. Stroke was defined as the new occurrence of a motor, sensory, or cognitive dysfunction which persists for more than 24 h.

### Statistical analysis

Descriptive analysis was performed and presented in the respective tables. Categorical variables are presented as number and percentages and continuous variables as mean ± standard deviation. Comparisons of the baseline clinical characteristics between the two age subgroups and between the two gender subgroups were performed using Pearson’s chi-squared test for categorical and the ANOVA test for continuous ones. The performance of the AUB-HAS2 Index within each age and gender subgroup was assessed by comparing the event rates among the different score groups (0, 1, 2, 3, and > 3). The Cochran-Armitage test for trend was used to evaluate the trend in the proportions of the outcome across the levels of the AUB-HAS2 score. Time to event analyses were carried out using the Kaplan-Meier curve, where significance was calculated using the log-rank test. Furthermore, receiver operating characteristic (ROC) curves were constructed, and the areas under the curve (AUC) were measured to assess the discriminatory power of the AUB-HAS2 Index in each subgroup. Comparison of AUCs of the AUB-HAS2 in the two age and gender subgroups was performed using the non-parametric *Z* test (DeLong et al., 1988). The Statistical Analysis Software (SAS, version 9.4) was used for data management and analyses. Statistical significance was set at the 0.05 alpha level.

## Results

### Age analysis

Table 2 shows a comparison of the baseline clinical and surgical characteristics between the two age subgroups. Patients ≥ 40 years old had a much higher prevalence of cardiovascular risk factors (other than smoking), comorbidities, prior history and symptoms of heart disease, and a higher American Society of Anesthesiology (ASA) class. Patients < 40 years old had more general surgery procedures, a higher proportion of emergency surgeries, and a higher percentage of operations done under general anesthesia.

**Table 2:** Clinical characteristics of the age and gender subgroups

	Total (*n* = 1,167,414)	Age ≥ 40 (*n* = 970,982)	Age < 40 (*n* = 196,432)	*P* value	Men (*n* = 492,686)	Women (*n* = 672,084)	*P* value
Demographics							
Age (years)	57 ± 17	62 ± 13	30 ± 6	–	59 ± 17	56 ± 17	< 0.0001
Male Gender	42	44	35	< 0.0001	–	–	–
Body mass index (kg/m^2^)	30 ± 8.4	30 ± 8.0	31 ± 10	< 0.0001	29 ± 7.2	31 ± 9	< 0.0001
CVD risk factors							
Diabetes mellitus	16.2	18.5	4.8	< 0.0001	18.3	14.7	< 0.0001
Hypertension	48.6	56.2	11.2	< 0.0001	53.0	45	< 0.0001
Current smoker	20.0	19.0	24.8	< 0.0001	22.6	18.1	< 0.0001
Cardiac history							
Myocardial infarction	0.6	0.7	0.1	< 0.0001	0.8	0.5	< 0.0001
Congestive heart failure	0.9	1.0	0.1	< 0.0001	1.1	0.7	< 0.0001
Previous coronary angioplasty	5.8	6.9	0.2	< 0.0001	8.9	3.5	< 0.0001
Previous cardiac surgery	5.8	6.9	0.5	< 0.0001	9.7	3.0	< 0.0001
Any history of heart disease	10.9	12.9	0.7	< 0.0001	16.9	6.4	< 0.0001
Symptoms of angina or dyspnea	10.4	11.6	4.7	< 0.0001	10.6	10.3	< 0.0001
Comorbidities							
Renal insufficiency (Cr > 1.5 mg/dl)	7.1	8.1	2.1	< 0.0001	10.3	4.7	< 0.0001
Dialysis	2.0	2.2	1.0	< 0.0001	2.6	1.5	< 0.0001
COPD	5.2	6.3	0.3	< 0.0001	6.3	4.5	< 0.0001
Stroke	4.5	5.4	0.5	< 0.0001	5.5	3.8	< 0.0001
Blood tests							
Hemoglobin (g/dl)	13 ± 1.8	13 ± 1.8	13.3 ± 1.7	< 0.0001	13.3 ± 1.9	12.7 ± 1.5	< 0.0001
Creatinine (mg/dl)	1.1 ± 1.0	1.1 ± 1.0	0.90 ± 0.90	< 0.0001	1.2 ± 1.1	0.9 ± 0.8	< 0.0001
Surgical characteristics							
Type of surgery							
General	64.4	61.7	77.8	< 0.0001	61.1	66.8	< 0.0001
Thoracic	1.0	1.1	0.6		1.3	0.8	
Orthopedic	8.7	9.6	4.2		8.8	8.6	
ENT (ear, nose, throat)	1.7	1.5	2.8		1.9	1.7	
Neurologic	2.6	2.7	2.1		3.1	2.2	
Gynecologic	4.8	4.4	6.9		0.0	8.3	
Urologic	3.7	4.2	1.1		6.8	1.4	
Plastic	1.4	1.3	1.9		0.6	1.9	
Vascular	11.7	13.5	2.7		16.4	8.2	
Anesthesia							
General	92.0	91.0	96.8	< 0.0001	91.2	92.5	< 0.0001
Spinal/epidural	3.1	3.6	0.7		3.6	2.8	
Regional	0.8	1.0	0.2		1.0	0.7	
Local	0.3	0.3	0.2		0.3	0.3	
Others	3.8	4.1	2.2		3.8	3.7	
American Society of Anesthesiology class							
1	7.8	4.4	24.8	< 0.0001	7.7	8.0	< 0.0001
2	44.1	41.7	55.6		38.5	48.2	
3	40.9	45.5	18.0		44.4	38.3	
> 3	7.2	8.3	1.6		9.5	5.5	
Emergency case	13.2	11.0	23.8	< 0.0001	15.1	11.7	< 0.0001
Total operation time (min)	114 ± 94	118 ± 96	92 ± 81	< 0.0001	119 ± 100	110 ± 89	< 0.0001
Hospital duration (days)	4.5 ± 9.4	4.8 ± 10.1	2.8 ± 8.1	< 0.0001	5.0 ± 10.1	4.0 ± 9.1	< 0.0001

Values are % or mean ± SD

*COPD* chronic obstructive pulmonary disease

**Fig. 1:**
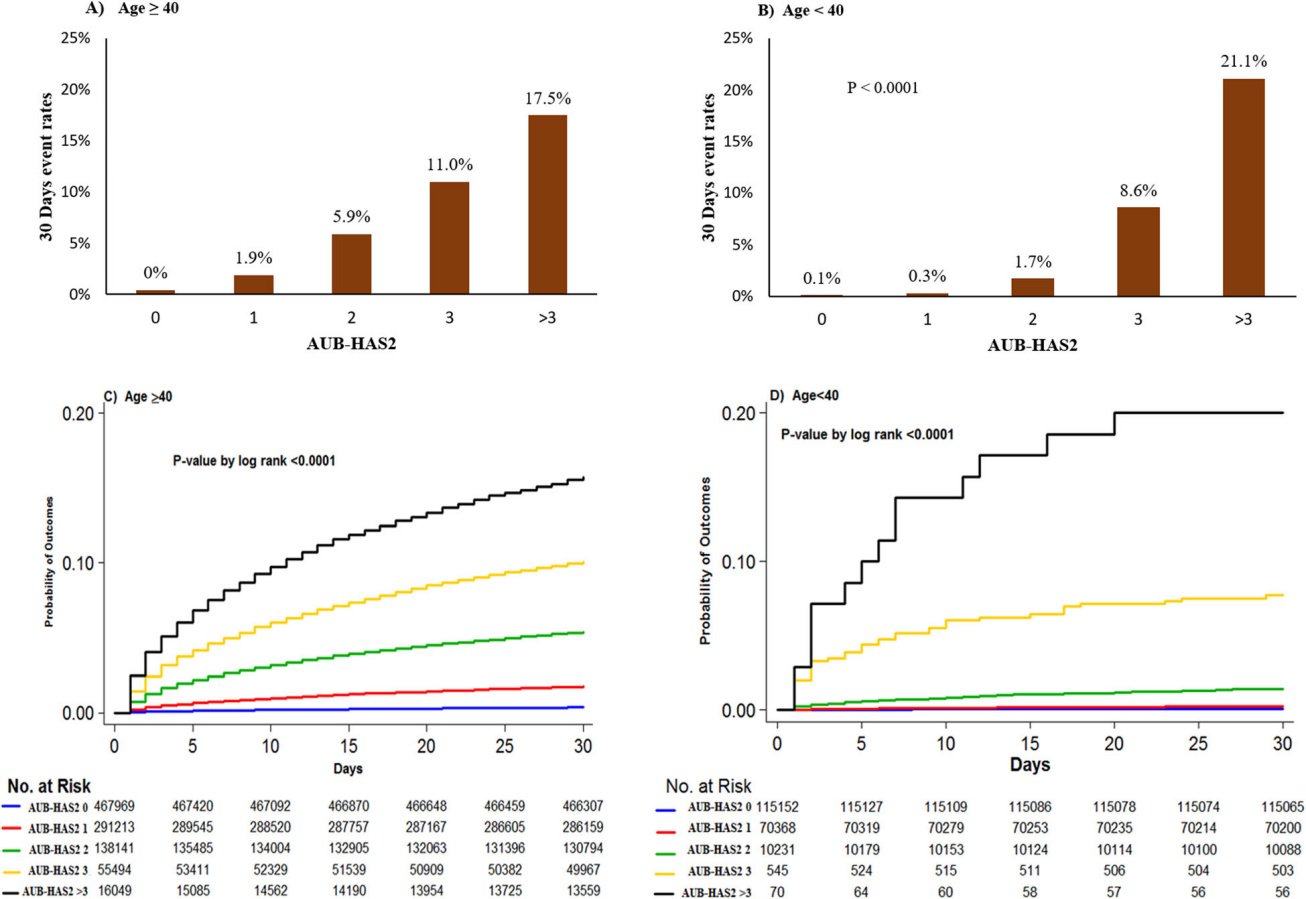
**A** and **B** Bar graphs comparing the 30-day event rates (death, myocardial infarction, or stroke) among the different AUB-HAS2 score groups in patients ≥ 40 and < 40 years old. In both age groups, there was a gradual increase in the event rates as the AUB-HAS2 score increased with a p value for trend of <0.0001. (**C** and **D**) Corresponding Kaplan-Meier curves that further illustrate the powerful discriminatory ability of the AUB-HAS2 index in stratifying peri-operative cardiovascular risk in both age subgroups

**Fig. 2:**
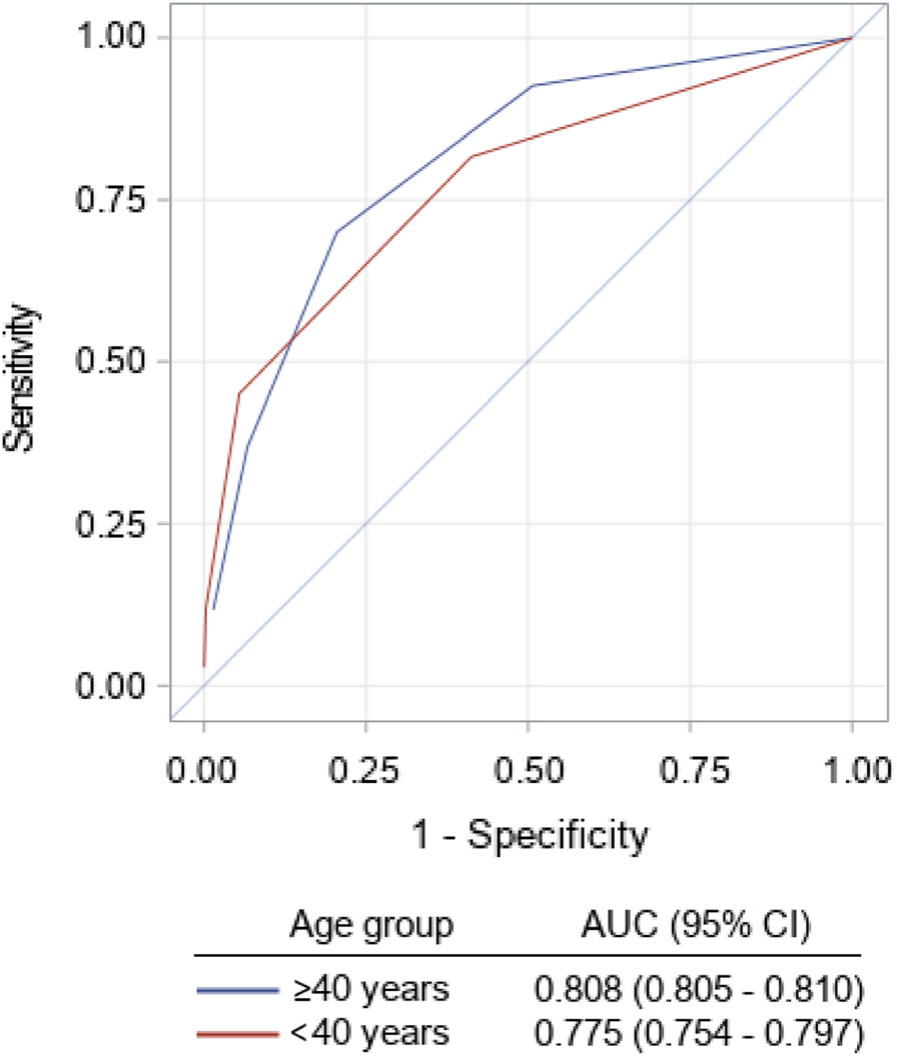
Plot of the receiver operating characteristic (ROC) curves of the AUB-HAS2 in the two age subgroups with the corresponding areas under the curve (AUC)

Table 3 and Fig. 1 compare the event rates among the different AUB-HAS2 score groups in each of the age strata. The overall 30-day event rate in patients ≥ 40 years was higher than that in patients < 40 years (2.5% vs 0.3%, *P* < 0.0001). In both age subgroups, there was a gradual and significant increase in the primary outcome measure (death, MI, or stroke) as the AUB-HAS2 score increased, from < 0.5% in those with a score of 0 to more than 15% in those with a score > 3. A similar trend was also present for each individual element of the outcome measure when measured separately. In patients ≥ 40 years, the AUB-HAS2 score was able to stratify risk into low (scores 0–1), intermediate (scores 2–3), and high (score > 3). Also, in patients < 40, the score was able to stratify risk into three risk groups, but here, a score of 2 had a relatively low risk of 1.7%. So, the categorization became low (scores 0–2), intermediate (score of 3), and high (score > 3). The stratification of risk in both age groups was further demonstrated by Kaplan-Meier survival curves as shown in Fig. 1. There was a gradual accumulation of events in patients ≥ 40 years over the 30-day follow-up period. Whereas in patients < 40 years old with a score of 3 or > 3, 80% of the events occurred within the first 2 weeks post-surgery. ROC curves (Fig. 2) showed the AUB-HAS2 Index has a very good discriminative power in both age strata with AUCs of 0.81 and 0.78, respectively.

**Table 3:** Comparison of outcomes among the AUB-HAS2 score groups within age and gender subgroups

	Total	AUB-HAS2 Score					
		0	1	2	3	> 3	***P*** value
**Age ≥ 40 (*n*)**	**970,982**	**468,094**	**291,617**	**138,803**	**56,073**	**16,395**	
Death, MI, or stroke	24,511 (2.5)	1813 (0.4)	5535 (1.9)	8124 (5.9)	6173 (11.0)	2866 (17.5)	< 0.0001
Death	18,574 (1.9)	1102 (0.2)	3931 (1.4)	6218 (4.5)	4949 (8.8)	2374 (14.5)	< 0.0001
MI	4758 (0.5)	425 (0.1)	1152 (0.4)	1551 (1.1)	1134 (2.0)	496 (3.0)	< 0.0001
Stroke	2969 (0.3)	395 (0.1)	802 (0.3)	919 (0.7)	602 (1.1)	251 (1.5)	< 0.0001
**Age < 40 (*n*)**	**196,432**	**115,161**	**70,390**	**10,260**	**550**	**71**	
Death, MI, or stroke	524 (0.3)	96 (0.1)	192 (0.3)	174 (1.7)	47 (8.6)	15 (21.1)	< 0.0001
Death	424 (0.2)	64 (0.1)	154 (0.2)	152 (1.5)	42 (7.6)	12 (16.9)	< 0.0001
MI	39 (0.0)	13 (0.0)	12 (0.0)	9 (0.1)	5 (0.9)	0 (0.0)	< 0.0001
Stroke	76 (0.0)	23 (0.0)	28 (0.0)	18 (0.2)	4 (0.7)	3 (4.2)	< 0.0001
**Males (*n*)**	***492,686***	***227,258***	***152,863***	***72,336***	***31,031***	***9,198***	
Death, MI, or stroke	13,158 (2.7)	1080 (0.5)	3042 (2.0)	4243 (5.9)	3227 (10.4)	1566 (17.0)	< 0.0001
Death	9882 (2.0)	685 (0.3)	2156 (1.4)	3204 (4.4)	2561 (8.3)	1276 (13.9)	< 0.0001
MI	2724 (0.6)	251 (0.1)	664 (0.4)	871 (1.2)	637 (2.1)	301 (3.3)	< 0.0001
Stroke	1509 (0.3)	215 (0.1)	405 (0.3)	454 (0.6)	305 (1.0)	130 (1.4)	< 0.0001
**Females (*n*)**	***672,084***	***354,686***	***208,337***	***76,369***	***25,464***	***7,228***	
Death, MI, or stroke	11,819 (1.8)	823 (0.2)	2674 (1.3)	4033 (5.3)	2983 (11.7)	1306 (18.1)	< 0.0001
Death	9069 (1.4)	477 (0.1)	1921 (0.9)	3148 (4.1)	2421 (9.5)	1102 (15.3)	< 0.0001
MI	2062 (0.3)	186 (0.1)	498 (0.2)	684 (0.9)	500 (2.0)	194 (2.7)	< 0.0001
Stroke	1532 (0.2)	201 (0.1)	424 (0.2)	483 (0.6)	301 (1.2)	123 (1.7)	< 0.0001

Values are *N* (%)

*MI* myocardial infarction

### Gender analysis

Table 2 shows a comparison of the baseline clinical and surgical characteristics between men and women. Men were older and had a higher prevalence of cardiovascular risk factors, comorbidities, prior history and symptoms of heart disease, and a higher ASA class. Men also had double the rate of vascular surgeries.

**Fig. 3:**
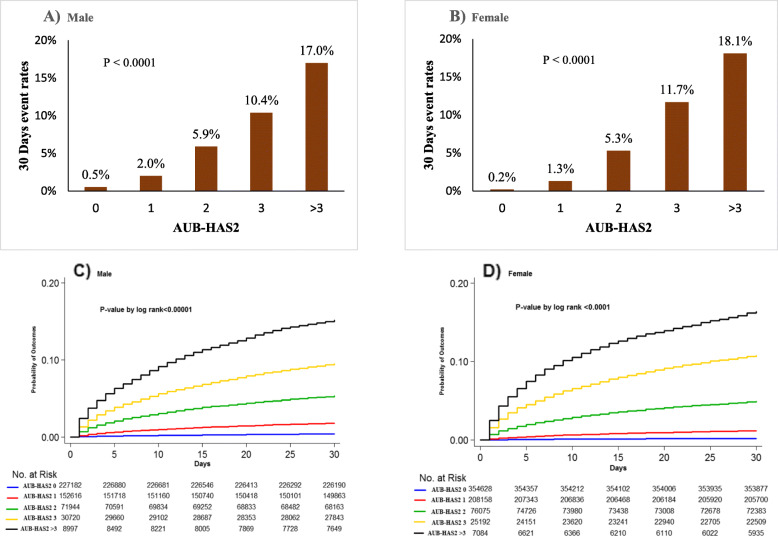
**A**, **B** Bar graphs comparing the 30-day event rates (death, myocardial infarction, or stroke) among the different AUB-HAS2 score groups in men and women. In both genders, there was a gradual increase in the event rates as the AUB-HAS2 score increased with a *P* value for trend of < 0.0001. **C**, **D** Corresponding Kaplan-Meier curves that further illustrate the powerful discriminatory ability of the AUB-HAS2 Index in stratifying peri-operative cardiovascular risk in both genders

**Fig. 4:**
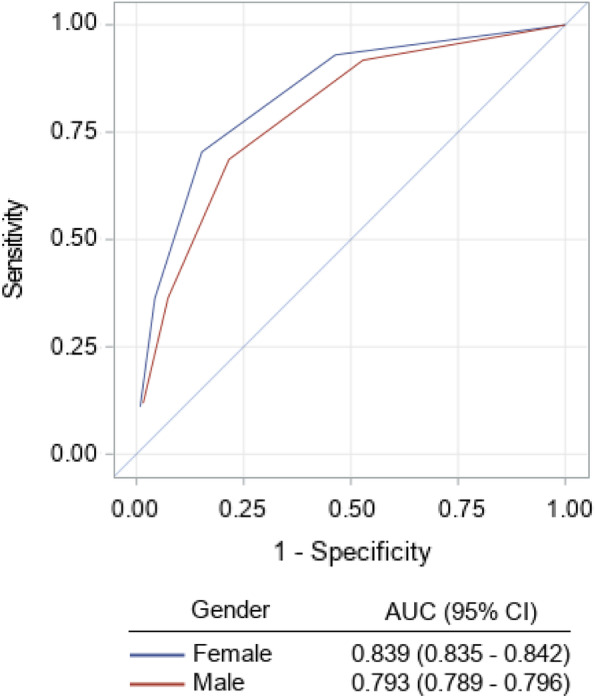
Plot of the receiver operating characteristic (ROC) curves of the AUB-HAS2 in the two gender subgroups with the corresponding areas under the curve (AUC)

Table 3 and Fig. 3 compare the event rates among the different AUB-HAS2 score groups in both genders. The overall 30-day event rate in men was higher than that in women (2.7% vs 1.8%, *P* < 0.0001). In both genders, there was a gradual and significant increase in the primary outcome measure (death, MI, or stroke) as the AUB-HAS2 score increased, from ≤ 0.5% in those with a score of 0 to more than 15% in those with a score > 3. A similar trend was also present for each individual element of the outcome measure when measured separately. In both men and women, the AUB-HAS2 score was able to stratify risk into low (scores 0–1), intermediate (scores 2–3), and high (score > 3). The stratification of risk in both genders was further demonstrated by Kaplan-Meier survival curves as shown in Fig. 3. ROC curves (Fig. 4) showed the AUB-HAS2 Index has a very good discriminatory power in both men and women with AUCs of 0.84 and 0.79, respectively.

## Discussion

Risk indices are important tools for the pre-operative cardiovascular assessment of patients scheduled for surgery. Their use is recommended by both US and European guidelines (Fleisher et al., 2014; Kristensen et al., 2014). Multiple testing of these indices in different patient populations is essential to establish their validity. The AUB-HAS2 Index was recently reported and validated in the general population of patients undergoing non-cardiac surgery (Dakik et al., 2019). We have recently added another level of validation of the index in a wide spectrum of surgical specialties and site-specific surgeries (Dakik et al., 2020a; Dakik et al., 2020b). This report adds yet another validation of this new index and underscores its power in the risk stratification of patients undergoing non-cardiac surgery across different age and gender subgroups.

Age is an important predictor for peri-operative cardiovascular events and is an essential element in most of the indices used for this assessment. The NSQIP MICA and ACS surgical calculator include age as a continuous variable in their predictive model (Gupta et al., 2011; Bilimoria et al., 2013). The RCRI, one of the early indices for pre-operative cardiovascular evaluation, does not include age as an element, but it was originally derived and validated in patients > 50 years old (Lee et al., 1999). The AUB-HAS2 Index was also derived from a population of patients older than 40 years old, but it further stratified patients using a cutoff age of 75 years and found that age > 75 years was an important variable in the multivariable regression analysis (Dakik et al., 2019). In the original study validating the AUB-HAS2 Index, all adult patients > 18 years old registered in NSQIP were utilized, and the index showed very good discriminative power. However, pre-operative cardiovascular evaluation is usually requested in routine clinical practice for adult patients who are more than 40 or 50 years old. This study was thus initiated to study the performance of the AUB-HAS2 Index in the two age groups ≥ 40 and < 40 years. The study confirms the very good discriminative power of the AUB-HAS2 Index in the subgroup of patients ≥ 40 years old who are registered in the large ACS NSQIP database, with an AUC of 0.81. It also further extends its validation to even younger patients (< 40 years) who generally do not undergo routine pre-operative cardiovascular evaluation. The AUB-HAS2 Index was able to stratify the risk very well in this subgroup as well with an AUC of 0.78. Patients < 40 years old with an AUB-HAS2 score of 3 or > 3 had event rates of 8.6% and 21.1%, respectively, compared to the very low event rate in those with a score < 3. Thus, although young patients do not routinely undergo pre-operative cardiovascular evaluation, this study suggests that particular attention should be given to those who have multiple elements of the AUB-HAS2 Index. Furthermore, and similar to our initial reports (Dakik et al., 2019; Dakik et al., 2020a), this new index was able to identify a large group of patients with a score of 0 who have a very low event rate (48% of patients > 40 years old and 59% of patients < 40 years old). This carries important logistic implications in that this large but low-risk group might not need routine pre-operative cardiovascular evaluation or special post-operative monitoring.

This study also extends the validation of the AUB-HAS2 Index as a powerful tool for pre-operative cardiovascular evaluation for both genders. Men had a higher risk profile than women in terms of risk factors and comorbidities, and this was associated with a higher 30-day event rate (2.7% vs 1.8%, *P* < 0.0001). Gender, however, did not remain a significant variable in the final multivariate logistic regression model of the AUB-HAS2 Index. It is also not an element in any of the other commonly used pre-operative risk indices. The AUB-HAS2 Index, however, persisted to be an important predictor of risk with a high discriminative power in both genders as shown in this study. Similar to the age subgroups, the AUB-HAS Index was also able to identify a large group of patients in both genders (46% of men and 53% of women) with a score of 0 who have a very low event rate of ≤ 0.5%, a finding that carries important practical implications for the triage of these patients and for the proper allocation of resources. Furthermore, and in both genders, there was a gradual increase in the events rates as the AUB-HAS2 score increased. The categorization of risk groups that was initially proposed in the derivation of this new index as low (scores 0–1), intermediate (scores 2–3), and high (> 3) was maintained in this study as well in both genders.

## Limitations

This study adds another level of validation of the AUB-HAS2 Index in age- and gender-specific subgroups in the ACS NSQIP database. However, although this is a large and multicenter database, it remains primarily representative of North America. The findings will need to be further validated in other patient populations in different countries with a wider geographic representation.

## Conclusion

This study extends the validation of the newly derived AUB-HAS2 cardiovascular risk index to different age and gender subgroups with very good discriminative power. Moreover, the AUB-HAS2 Index was able to identify, in all of these subgroups, a large group of low-risk patients with a score of 0 who, in general, might not require any special pre-operative cardiovascular evaluation or post-operative monitoring.

## Data Availability

All data generated or analyzed during this study are included in this published article.
